# The dissection of transcriptional modules regulated by various drugs of abuse in the mouse striatum

**DOI:** 10.1186/gb-2010-11-5-r48

**Published:** 2010-05-04

**Authors:** Marcin Piechota, Michal Korostynski, Wojciech Solecki, Agnieszka Gieryk, Michal Slezak, Wiktor Bilecki, Barbara Ziolkowska, Elzbieta Kostrzewa, Iwona Cymerman, Lukasz Swiech, Jacek Jaworski, Ryszard Przewlocki

**Affiliations:** 1 Department of Molecular Neuropharmacology, Institute of Pharmacology PAS, Smetna 12, Krakow, 31-343, Poland; 2 Laboratory of Molecular and Cellular Neurobiology, International Institute of Molecular and Cell Biology, Ks. Trojdena 4, Warsaw, 02-109, Poland

## Abstract

**Background:**

Various drugs of abuse activate intracellular pathways in the brain reward system. These pathways regulate the expression of genes that are essential to the development of addiction. To reveal genes common and distinct for different classes of drugs of abuse, we compared the effects of nicotine, ethanol, cocaine, morphine, heroin and methamphetamine on gene expression profiles in the mouse striatum.

**Results:**

We applied whole-genome microarray profiling to evaluate detailed time-courses (1, 2, 4 and 8 hours) of transcriptome alterations following acute drug administration in mice. We identified 42 drug-responsive genes that were segregated into two main transcriptional modules. The first module consisted of activity-dependent transcripts (including *Fos *and *Npas4*), which are induced by psychostimulants and opioids. The second group of genes (including *Fkbp5 *and *S3-12*), which are controlled, in part, by the release of steroid hormones, was strongly activated by ethanol and opioids. Using pharmacological tools, we were able to inhibit the induction of particular modules of drug-related genomic profiles. We selected a subset of genes for validation by *in situ *hybridization and quantitative PCR. We also showed that knockdown of the drug-responsive genes *Sgk1 *and *Tsc22d3 *resulted in alterations to dendritic spines in mice, possibly reflecting an altered potential for plastic changes.

**Conclusions:**

Our study identified modules of drug-induced genes that share functional relationships. These genes may play a critical role in the early stages of addiction.

## Abstract 2

The transcriptional response to six commonly-abused drugs was assessed in the mouse brain revealing common modules of drug-induced genes.

## Background

Drug addiction is a brain disease with prominent hazardous effects, including the collapse of health and social and economic status [1]. Acute exposure to drugs of abuse initiates molecular and cellular alterations in the central nervous system [2,3] that lead to an increased overall vulnerability to addiction with subsequent drug exposures [4]. These drug-induced alterations employ changes in gene transcription that result in the synthesis of new proteins [5]. Therefore, one of the important goals of addiction research is to identify the drug-induced gene expression changes in the specific brain structures that are related to the addictive properties of various drugs.

The major neural target sites of addictive drugs are the ventral and the dorsal striatum, that is, the brain regions that control reward sensitivity, motor function and habit learning [6]. The dorsal striatum is thought to underlie stimulus-response and spatial learning, and the ventral striatum is involved in appetitive behavior and reinforcement [7,8]. However, to some extent, these functions might overlap [9,10]. All addictive drugs elevate dopamine levels in the striatum, and this effect is associated with reinforcing drug properties [11]. However, the pharmacological mechanisms and neural substrates involved in mediating the rewarding action are different for various drugs. Psychostimulants directly influence extracellular dopamine levels in the striatum through inhibitory effects on dopamine reuptake [12,13]. Opiates inhibit GABAergic inhibitory neurons in the ventral tegmental area and activate dopaminergic neurons projecting to the striatum [14]. In addition, opiates directly bind to opioid receptors located on striatal interneurons [15]. Ethanol acts on GABAergic interneurons in the ventral tegmental area that, in turn, modulate the activity of dopaminergic neurons and the action of neurotransmitter-gated ion channels [16]. Nicotine enhances reward-related dopamine release by activating nicotinic acetylcholine receptors [17,18]. Therefore, it is believed that the combination of dopamine-dependent neurotransmission and endogenous opioid-dependent modulation is responsible for the acquisition of drug addiction [4,19]. The molecular and genomic mechanisms by which drugs of abuse induce neuroplastic changes related to addiction remain largely unknown [20].

Several studies have evaluated changes in gene expression profiles in the brain after administration of drugs of abuse (reviewed in [21]). Exposure to psychostimulants induces the activity-dependent gene expression of several transcription activators and repressors [22,23]. Opioids and ethanol regulate the transcription of genes involved in metabolic functions and a group of genes encoding heat-shock proteins [24-28].

Genomic research strategies have recently transitioned from the search for unknown genes to the identification and evaluation of coordinated gene networks and transcriptional signatures [29]. New opportunities arising from the analysis of these networks include identifying novel relationships between genes and signaling pathways, connecting biological processes with the regulation of gene transcription, and associating genes and gene expression with diseases [30,31]. The identification of gene networks requires large gene expression data sets with multiple data points [32]. The transcriptional response to a drug treatment analyzed during a time-course suits the above strategy perfectly.

Exploring dynamic changes in brain gene expression profiles is possible only in animal models. In these models, assessments of the behavioral effects of drugs of abuse are well established. Therefore, integrating brain gene transcription and phenotypic information provides us with a unique opportunity to associate the addictive potential of the drugs with the molecular responses activated by these drugs [33,34]. The limitations of such a strategy include differences in drug responses between humans and rodents and the extreme complexity of the analyzed tissue. Despite these limitations, the obtained results may provide new insights into the molecular control of drug addiction.

In this study, we aimed to identify the transcriptional networks activated by different classes of addictive drugs and to translate the gene expression patterns into biological themes that are related to the development of addiction.

## Results

### Comparison of rewarding and stimulant drug properties

**Figure 1:**
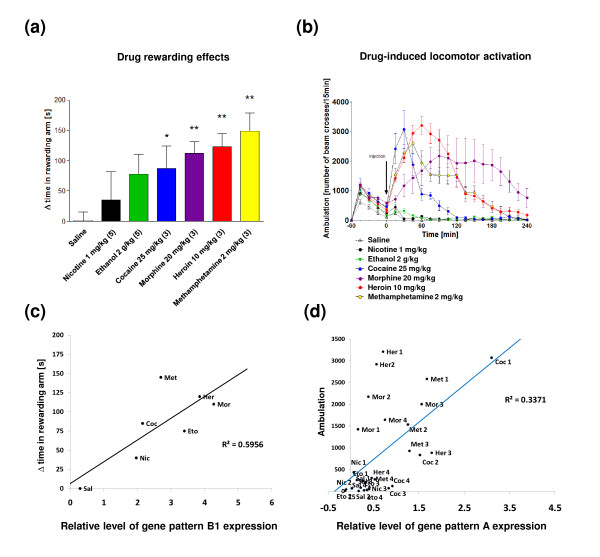
**Comparison of the reinforcing and activating effects of drugs of abuse in C57BL/6J mice**. **(a) **Bar graph summarizing the development of CPP to morphine, heroin, ethanol, nicotine, methamphetamine, cocaine or saline injections (i.p.). The number of drug conditioning sessions is indicated in parentheses. The level of significance was measured using ANOVA following the Newman-Keuls post-hoc test for drug versus saline; *P < 0.05; **P < 0.01 (n = 6 to 12). **(b) **Graph summarizing locomotor activation in response to drug treatment measured as increased ambulation in an activity cage during 4 h (n = 5). **(c,d) **Analysis of correlations between drug-induced changes in gene expression and behavioral effects of drugs in mice (Additional file 9). Scatter plots present the most significant correlation between the behavioral effects (y-axis) and the level of drug-induced transcription (x-axis). Correlation with locomotor activation was computed using data for each particular time point.

In the present study, we assessed the behavioral and transcriptional effects of cocaine (25 mg/kg intraperitoneally (i.p.)), methamphetamine (2 mg/kg i.p.), morphine (20 mg/kg i.p.), heroin (10 mg/kg i.p.), ethanol (2 g/kg i.p.) and nicotine (1 mg/kg i.p.) on C57BL/6J mice. Drug doses previously reported to generate rewarding and addictive responses in mice were selected [35-37]. The rewarding properties were compared in our laboratory. Conditioned place preference (CPP) tests were performed using an unbiased procedure in a three-arm apparatus. Cocaine, morphine, heroin and methamphetamine treatment induced a robust preference for the drug-paired compartment (ANOVA, Newman-Keuls test, *P *< 0.05; Figure 1a). For ethanol and nicotine, the procedure was increased to five sessions of conditioning. Ethanol treatment induced a moderate effect in the CPP test (*t*-test, *P *< 0.05). Nicotine treatment produced a tendency for place preference, which may be associated with a very narrow effective dose range for reinforcing the effects of nicotine in mice.

An independent group of animals was tested for drug-induced motor behavior. Cocaine, methamphetamine, heroin and morphine treatment significantly increased locomotor activity following acute drug administration (repeated-measures ANOVA, Newman-Keuls test, *P *< 0.05; Figure 1b). Ethanol and nicotine treatment did not produce locomotor activation in comparison to saline-treated controls. The behavioral data were further used to analyze associations between phenotypes and transcriptome alterations.

### Whole-genome gene expression profiling

We applied a strategy of detailed time-course studies of gene expression alterations following acute administration of various drugs of abuse using Illumina Whole-Genome 6 microarrays. To analyze the dynamics of early, intermediate and relatively late changes in mRNA abundance, the analysis was performed at four time points (1, 2, 4 and 8 h following drug injection).

**Figure 2:**
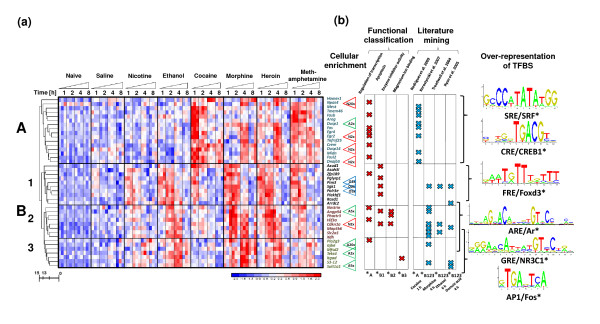
**Hierarchical clustering of drug-dependent transcriptional alterations in mouse striatum**. **(a) **Microarray results are shown as a heat map and include genes with a genome-wide significance from two-way ANOVA of the drug factor. Colored rectangles represent transcript abundance (Additional file 2) 1, 2, 4 and 8 h after injection of the drug indicated above of the gene labeled on the right. The intensity of the color is proportional to the standardized values (between -2 and 2) from each microarray, as indicated on the bar below the heat map image. Clustering was performed using Euclidean distance according to the scale on the left. Major drug-responsive gene transcription patterns are arbitrarily described as 'A', 'B1', 'B2' and 'B3. **(b) **Gene cluster analysis using data-mining methods (Table 1). The fold cellular enrichment (2, 5 or 20 in a particular cell population, as reported in Cahoy *et al*. [101]) of the selected transcripts in various cell types is indicated by N (neurons), A (astrocytes) or O (oligodendrocytes). Over-representation of transcription factor binding site (TFBSs), as indicated on the left, was identified using the cREMaG database (see Materials and methods). The statistical significance of enrichment is marked as *P < 0.05.

Microarray data analysis using two-way ANOVA identified 42 drug-responsive genes with *P *< 1 × 10^-6 ^(corresponding to *P *< 0.05 after adjusting for approximately 48,000 independent tests using Bonferroni correction; Figure 2). Compared to other gene expression profiling studies, the statistical threshold was rather conservative. However, the same threshold is widely accepted in population genetic and genome-wide association studies in humans [38]. The difference between the methodological standards may result from the number of samples and biological replicates usually used in these two types of whole-genome studies. The present study contained (relatively) many high-quality samples, allowing it to satisfy restrictive statistical criteria.

**Table 1:** Functional classes, results from literature, and transcription factor binding sites associated with drug-regulated patterns of gene expression

Gene pattern	Gene ontology		Literature mining		TFBS over-representation	
			
	Term	Fold (*P*)^a^	Dataset	*P* ^a^	Binding sites^b^	Fold (*P*)^c^
A	Protein phosphatase activity	32.4 (0.0036)	Rodriguez *et al*. [108], cocaine 1 h	1.33E-36	SRF (MA0083)	5.7 (0.095)
	Rhythmic process	14.7 (0.0166)	Jayanthi *et al*. [40], methampethamine 2 h	1.04E-13	CREB1 (MA0018)	3.9 (0.0068)
	Phosphotransferase activity	10.7(<0.0001)	Lemberger *et al*. [41], kainic acid 1 h	5.88E-13		
	Protein dimerization activity	3.6 (0.0203)	Ryan *et al*. [42], domoic acid 0.5 and 1 h	3.52E-12		
	Regulation of transcription	3 (0.0001)	Impey *et al*. [43], forskolin 1 h	3.87E-12		
						
B_1_	Small GTPase mediated signal transduction	5.9 (0.0085)	Sanchis-Segura *et al*. [44], morphine 4 h	8.96E-29	Foxd3 (MA0041)	4.4 (0.02)
	Apoptosis	5 (0.0018)	Treadwell *et al*. [46], ethanol 6 h	1.87E-11	Foxa2 (MA0047)	4.2 (0.043)
	Cell cycle	4.7 (0.0025)	Sato *et al*. [47], dexamethasone 2 h	1.57E-08	FOXF2 (MA0030)	4 (0.025)
	Intracellular signaling cascade	3.2 (0.0079)	Lemberger *et al*. [41], kainic acid 1 h	9.51E-07	Evi1 (MA0029)	3.8 (0.036)
	Intracellular	1.5 (0.0017)	Ryan *et al*. [42], domoic acid 4 h	8.90E-07		
						
B_2_	Enzyme inhibitor activity	8.9 (0.041)	Korostynski *et al*. [45], morphine 4 h	1.15E-29	NR1H2 (MA0115)	3.5 (0.288)
	Apoptosis	5.9 (0.0007)	Ryan *et al*. [42], domoic acid 4 h	1.41E-18	Ar (MA0007)	3.3 (0.074)
	Response to stress	4.2 (0.01)	McClung *et al*. [27], morphine withdrawal	4.23E-17	NR2F1 (MA0017)	3.3 (0.0428)
	Cell differentiation	2.5 (0.026)	Treadwell *et al*. [46], ethanol 6 h	3.58E-13		
	Intracellular	1.4 (0.0074)	Chen *et al*. [109], heart failure left ventricular assist device (LVAD)	1.47E-07		
						
B_3_	Regulation of developmental process	9.1 (0.0364)	Korostynski *et al*. [45], morphine 4 h	3.75E-15	Fos (MA0099)	6.7 (0.0103)
	Magnesium ion binding	8.5 (0.0416)	McClung *et al*. [27], morphine withdrawal	3.52E-10	NR3C1 (MA0113)	5.6 (0.0058)
	Anatomical structure morphogenesis	3.9 (0.0261)	Ryan *et al*. [42], domoic acid 4 h	3.22E-08	Ar (MA0007)	4.7 (0.0021)
	Calcium ion binding	3.5 (0.1894)	Treadwell *et al*. [46], ethanol 6 h	9.56E-06	TEAD1 (MA0090)	3.9 (0.0302)
	Transmembrane transporter activity	3.4 (0.1973)	Hasan *et al*. [110], chronic oxycodone	7.61E-05		

The complete results of data-mining are presented in Additional files 7 and 8. ^a^Statistical significance of gene enrichment was computed using algorithms implemented in DAVID 2008 database and ErmineJ software. ^b^Conserved promoter regions +5,000/-1,000 bp from the transcription start site were analyzed (see Materials and methods). ^c^Fold change of the detected number of identified transcription factor binding sites (TFBSs) compared to the number expected by chance. ^d^Statistical significance of over-representation of TFBS-containing genes compared to a number expected by chance was computed using a *z*-score test.

Furthermore, we estimated the false discovery rate (percent FDR) to answer the question of how large was the fraction of drug-responsive genes discovered at the assumed threshold [39]. The maximum number of true positive genes altered in the striatum by drugs of abuse (drug factor, 104 transcripts) was found at a 29% FDR. Beyond that level, the number of true positives did not increase. Surprisingly, the number of true positives remained stable (84 to 104 transcripts, mean = 94.4 ± 4.9) over a wide range of FDR (4.7 to 56.3%). The results for the drug factor are in contrast to alterations in the striatal gene expression profile related to the time point of the experiment (time factor). The maximum number of true positive genes (5,442 transcripts) for the time factor was found at a 69.8% FDR and increased linearly in the range 0.1 to 69.8% FDR (Additional file 1).

The above observations suggest a rather unexpected conclusion. While the diurnal cycle alters a vast fraction of the brain transcriptome, drugs regulated the expression of a limited number of genes (approximately 100), and this alteration was robust. The number of genes obtained using Bonferroni correction (42 transcripts) was equal to the number of genes obtained at a 0.1% FDR threshold. Therefore, at the chosen threshold, we identified 40.3% (42 of 104 transcripts) of genes altered by drugs of abuse with 99.9% confidence. The complete results of the ANOVA, including two different methods of correction for multiple comparisons (Bonferroni correction and percent FDR) for both time and drug factors are provided in Additional file 2.

**Figure 3:**
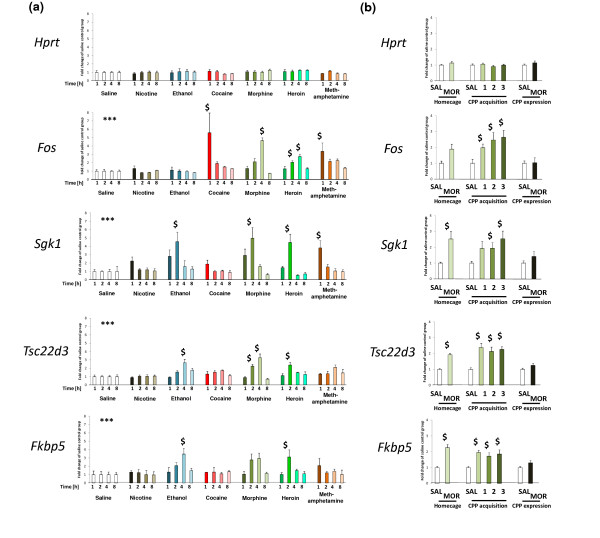
**Validation of drug-induced regulation of gene expression**. **(a) **Bar graphs summarizing qPCR-based measurement of changes in selected gene expression after the indicated drug injection, presented as fold change over the saline control group with standard error (n = 5 to 6). Significant differences in the main effects from multivariate ANOVA for drug treatment are indicated by asterisks (***P < 0.001) and from the Bonferroni post-hoc test (versus appropriate saline control) by dollar signs (*P *< 0.05). **(b) **Bar graphs summarizing qPCR-based measurement of selected gene expression after morphine (MOR) injection in the home cage or during CPP acquisition and expression. Results are presented as fold change over the saline control group (SAL) with standard error (n = 6 to 7). Significant differences in transcript abundance between the morphine-treated and control animals obtained by a *t*-test are indicated by dollar signs (*P *< 0.05).

The changes in mRNA abundance of selected marker genes were validated by quantitative PCR (qPCR) using aliquots of the non-pooled total RNA (Figure 3a; Additional file 3), yielding an overall correlation between the microarray and qPCR results of r = 0.69 (Spearman's method, *P *= 4.87 × 10^-24^). The alterations in mRNA level were also confirmed in an independent experiment. In addition, the expression of the selected genes was evaluated during the acquisition and expression of morphine-induced CPP (Figure 3b).

### Identification of drug-regulated gene expression patterns

Hierarchical clustering revealed two major drug-responsive gene transcription patterns (arbitrarily described as A and B; Figure 2a). Pattern B consisted of three subsequent subclusters (arbitrarily described as B_1_, B_2 _and B_3_). Example genes from the particular patterns include: pattern A, *Fos*, *Egr2 *and *Homer1; *pattern B_1_, *Sgk1*, *Plekhf1 *and *Rasd1*; pattern B_2_, *Tsc22d3*, *Cdkn1a *and *Map3k6*; and pattern B_3_, *Fkbp5*, *S3-12 *and *Sult1a1*.

To search for other drug-responsive transcriptional networks, we performed additional hierarchical clustering on the lists of genes obtained with 5% (a threshold commonly used in the literature) and 29% (the maximum number of true positive results) FDRs. With these less restrictive statistical criteria, we found no other networks with distinct gene expression profiles (Additional file 4). Assuming that we identified all the major gene patterns altered by drugs of abuse and taking into consideration the fact that Bonferroni correction for multiple testing is very conservative, lists of genes with a significant level of Pearson's correlation to the main clusters (*P *< 1 × 10^-10^; FDR for this analysis was estimated at <0.1%) were extracted and analyzed.

Gene Ontology (GO) enrichment, literature mining and identification of *cis*-regulatory elements was performed on extended lists of transcripts from each gene expression pattern: A, 38 genes; B_1_, 45 genes; B_2_, 31 genes; and B_3_, 18 genes. Due to the similar profiles of the B_1 _and B_2_, as well as the B_2 _and B_3_, gene subsets, the lists partially overlapped by 27% and 45%, respectively (Additional file 2). One gene (*Car12*) was excluded from the analysis due to its outlying gene expression profile.

The drug-responsive genes are randomly distributed throughout the entire mouse genome. Chromosome localizations are shown in Additional file 5.

### Comparison of drug effects on the striatal transcriptome

The results of gene expression profiling revealed differences and similarities in the transcriptional responses to the various drugs (Figure 2; Additional file 6). Pattern A was induced 1 to 2 h after injection of cocaine or methamphetamine and 4 h after injection of morphine or heroin. Pattern B consisted of three subsequent subclusters: B_1_, induced 1 to 2 h after injection of ethanol, morphine, heroin, methamphetamine and cocaine; B_2_, induced 2 to 4 h after injection of ethanol, morphine, heroin and methamphetamine; and B_3_, induced 4 h after injection of ethanol, morphine and heroin. The only pattern common to all inspected drugs was pattern B_1_. However, this pattern was induced by different drugs to different degrees.

The drugs were divided into two groups. One, including cocaine and methamphetamine, exhibited high and early induction of pattern A and low or absent induction of pattern B. The second group, including ethanol, morphine and heroin, elicited high induction of pattern B. The complete results of a Tukey's post-hoc test (*P *< 0.05, drug versus saline) after ANOVA are provided in Additional file 2.

### Functional classification of drug-responsive genes

To identify functional associations between the genes with expression induced by drugs, we used three different data-mining tools (Figure 2b, Table 1). To characterize the transcriptional representation of biological processes, a list of genes from each gene expression pattern was analyzed by GO. Among the most abundant group of genes in pattern A, functional clusters of transcripts connected with protein phosphatase activity (32.4-fold enrichment, *P *< 0.01; for example, *Dusp1, Dusp6*), rhythmic processes (14.7-fold, *P *< 0.05; for example, *Per1, Per2*) and transcriptional regulatory activity (3-fold, *P *< 0.001; for example, *Fos, Egr2*) were over-represented.

The group of genes from pattern B_1 _was enriched in transcripts involved in small GTPase-mediated signal transduction (5.9-fold, *P *< 0.01; for example, *Rhou, Rasd1*), apoptosis (5-fold, *P *< 0.01; for example, *Gadd45 g, Sgk1*) and the cell cycle (4.7-fold, *P *< 0.01; for example, *Gadd45 g, Nedd9*). Analysis of pattern B_2 _revealed the enrichment of genes connected to enzyme inhibitor activity (8.9-fold, *P *< 0.05; for example, *Cdkn1a, Angptl4*), the stress response (4.2-fold, *P *< 0.01; for example, *Cdkn1a, Tsc22d3*) and regulation of cell differentiation (2.5-fold, *P *< 0.05; for example, *Plekhf1, Zbtb16*). Over-representation of transcripts involved in magnesium ion binding (8.5-fold, *P *< 0.05; for example, *Itgad, Atp10a*) was observed within gene expression pattern B_3_. A detailed description of the results of GO classification is included in Additional file 7.

### Comparison with previously reported gene expression profiles

To find points of reference for our results, we compared the lists of genes from the co-expressed gene patterns with previously described changes in gene expression profiles. Literature mining was based on the lists of genes reported as regulated in published manuscripts or found in publicly available datasets. Overall, we compared our data with 1,267 gene sets (Additional file 8).

We found high similarity with pattern A to lists of genes regulated following cocaine (*P *= 1.33 × 10^-36^) and methamphetamine (*P *= 1.04 × 10^-13^, FDR-corrected) administration [40]. Moreover, significant enrichment of genes regulated by kainic acid (*P *= 5.88 × 10^-13^) and domoic acid (*P *= 3.52 × 10^-12^) in the brain and by forskolin (*P *= 3.87 × 10^-12^) *in vitro *was also found in this group [41-44]. All of these *in vivo *effects were observed at a relatively early time point (1 to 2 h after injection) and were connected with the induction of a group of immediate early gene (IEG) transcription factors and neuroplasticity-related genes like *Fos*, *Arc*, *Npas4 *and *Homer1*.

Drug-induced transcription pattern B revealed different links with the published gene expression profiles than gene pattern A. The effects of morphine (*P *= 1.15 × 10^-29^) and ethanol (*P *= 1.87 × 10^-11^) on the activation of gene expression pattern B were in agreement with previous results [45,46]. These genes were induced between 2 and 4 h following drug injection. The regulation of gene expression pattern B was somewhat similar to the effects of the glucocorticoid receptor (GR) agonist dexamethasone in the hypothalamus (*P *= 1.57 × 10^-8^) [47]. Moreover, expression pattern B contained genes reported to be up-regulated in response to domoic acid (*P *= 1.41 × 10^-18^) at a relatively late time point (4 h after injection; Table 1) [42].

We also found that gene expression pattern A was similar to the group of dopamine receptor 1 (D1R) antagonist (SCH23390)-sensitive methamphetamine-responsive genes (*P *= 1.05 × 10^-6^). In contrast, pattern B_1 _was similar to the group of SCH23390-resistant methamphetamine-responsive genes (*P *= 4.20 × 10^-5^) [40]. A detailed description of the results of literature mining is included in Additional file 8.

### Identification of transcription factor binding sites

To discover molecular factors that are involved in the transcriptional control of the discovered gene expression patterns, we used an *in silico *method of transcription factor binding site (TFBS) identification. We analyzed gene promoters based on the assumption that a subset of the co-expressed genes may be co-regulated by common transcription factors. For this purpose, we developed a new tool for the discovery of over-represented TFBSs: the cREMaG database (see Materials and methods).

Gene expression pattern A revealed the highest over-representation of binding sites for serum response factor (SRF)/serum-responsive elements (5.7-fold higher than expected by chance, *P *= 9.5 × 10^-3^). Significant over-representation of TFBSs for transcription factor cyclic AMP-response element binding protein (CREB)/cyclic AMP response elements (3.9-fold, *P *= 6.8 × 10^-3^) was also found. We identified an over-representation of cyclic AMP response elements (the binding site for the CREB transcription factor) and serum-responsive elements (the binding site for the SRF transcription factor) in the core promoter regions of genes with expression pattern A. The complementary roles of these transcription factors have been independently confirmed [41,48].

The analysis of the promoter regions of pattern B_1 _genes indicated an over-representation of the binding site for transcription factors of the FOX family, Foxd3/FRE (4.4-fold, *P *= 1.96 × 10^-2^). Forkhead transcription factors are implicated in the neuronal response to oxidative stress [49]. Promoter regions of genes from expression pattern B_2 _contained relatively more binding sites for steroid hormones NR1H2-RAXR (3.5-fold, *P *= 2.88 × 10^-2^) and Ar/ARE (3.3-fold, *P *= 7.4 × 10^-2^) with transcriptional activity. An enrichment of binding sites for nuclear receptors in promoter regions, including the androgen receptor (ARE/Ar), was found, suggesting that genes from this subgroup may be regulated by steroid hormones. Over-representation of binding sites for Fos/AP1 (6.7-fold, *P *= 1.03 × 10^-2^) and NR3C1/GRE (5.6-fold, *P *= 5.8 × 10^-3^) was observed within promoter regions of genes from pattern B_3 _(Table 1). Components of the transcriptional complex AP-1 (*Fos*, *Fosb*) exhibited gene expression pattern A. Therefore, the occurrence of an AP-1 site in the promoter regions of genes expressed relatively late following drug administration may indicate target genes for the drug-activated transcription factors. The second putative mechanism of B_3 _gene regulation is related to the effects of glucocorticoid hormones on the central nervous system.

### Pharmacological dissection of drug-regulated gene patterns

**Figure 4:**
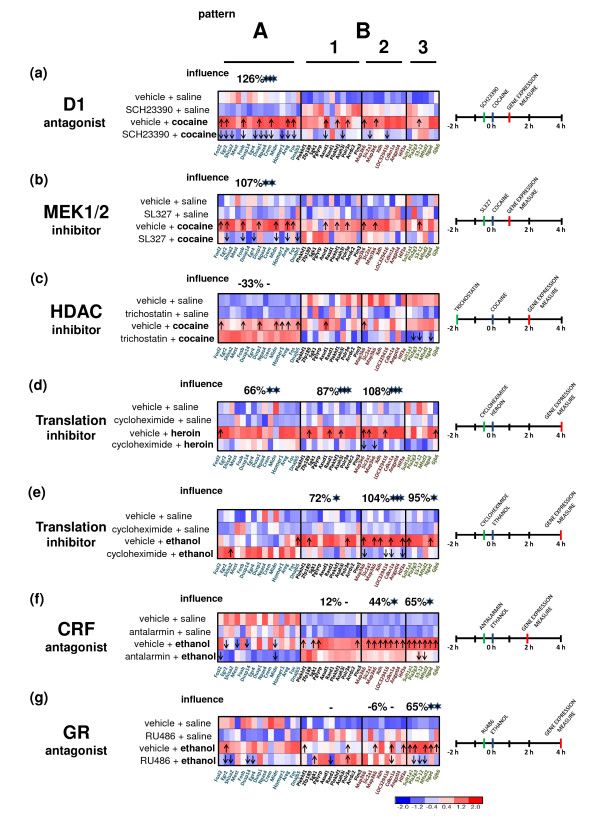
**Pharmacological dissection of transcriptional networks from the drug-induced gene expression profile**. Microarray results are shown as heat maps that include drug-responsive genes with genome-wide significance (Figure 2a). Colored rectangles represent transcript abundance and are labeled below the heat map. Each row contains the mean value from three independent array replicates, where samples from two mice were pooled and used for each microarray. The intensity of the color is proportional to the standardized values (between -2 and 2) from each microarray, as indicated on the bar below the cluster images. The names of enzyme inhibitors or receptor antagonists (inhibitor/antagonist) are indicated on the left. The time scheme of each experiment **(a-g) **is presented on the right. The arrow indicates (two-tailed *t*-test, *P *< 0.05) up- or down-regulation of the expression of a particular gene in comparisons between the drug plus vehicle and saline plus vehicle groups (upper row on each heat map) or drug plus inhibitor/antagonist and drug plus vehicle groups (bottom row). The overall influence was measured as a percentage of inhibition of the drug-induced transcriptional response, with 0% representing no effect and 100% representing complete inhibition. The statistical significance of influence was measured as a comparison of the mean fold change between the drug plus inhibitor/antagonist and saline plus vehicle versus drug plus vehicle and saline plus vehicle groups. The level of significance was measured using a two-tailed *t*-test: *P < 0.05; **P < 0.01; ***P < 0.001. CRF, corticotrophin-releasing factor; HDAC, histone deacetylase.

The effects of selected pharmacological tools on drug-induced gene expression changes were analyzed using DNA microarrays. This novel approach allowed us to modulate the drug-induced gene transcription and to dissect the particular genetic networks. Based on the results of the primary microarray experiment, six potential mechanisms of gene regulation were tested (Figure 4). However, due to an increase in the number of factors (various inhibitors and vehicles), it was not possible to perform all experiments in the time-course. Therefore, for each analysis, a drug and a time point identified in the first experiment as producing the maximal transcriptional effect were chosen (Additional file 6). Taking into account previously suggested anti-addictive properties of the substances that attenuate gene expression patterns [50-53], these results are important for further studies of potential therapeutic drugs.

The selected regulatory processes were tested for their influence on drug-induced gene expression pattern A. Pre-treatment with a D1R antagonist (SCH23390) blocked drug-induced CREB1/SRF-mediated gene transcription in the striatum, with a 126% reduction (26% of induction below basal level) in the level of cocaine activation (*P *< 0.001) (Figure 4a). This observation suggested that the regulatory intracellular cascades are activated mainly in striatal cells containing D1R. At the same time, SL327, an inhibitor of extracellular signal-regulated kinase (ERK1/2) activator kinase MEK1/2, inhibited the cocaine-activated expression of genes from pattern A, with a 107% reduction (7% of induction below basal level) in the level of cocaine activation (*P *< 0.001) (Figure 4b). This observation clearly indicates the involvement of the ERK1/2 signaling pathway [50].

Moreover, the administration of the histone deacetylase inhibitor trichostatin before cocaine administration provoked an intensification of the transcriptional response, with a 33% increase in the level of cocaine activation (Figure 4c). This observation suggested that the induction of genes from the expression pattern A may require enhanced chromatin unfolding [54].

Opioids increased the abundance of mRNAs from expression pattern A 4 h after injection. Pretreatment with the protein synthesis inhibitor cycloheximide (CHX) inhibited this induction, with a 66% reduction in the level of heroin activation (*P *< 0.05) (Figure 4d), which indicates that this relatively late response depends on protein translation.

All of the compared drugs induced gene transcription pattern B_1 _in the striatum. The relatively early transcriptional response to heroin (CHX blocked 87% of activation, *P *< 0.001) and ethanol (CHX blocked 72% of activation, *P *< 0.05) was blocked by an inhibitor of protein synthesis (Figure 4d, e). Similar effects of CHX were observed on the induction of gene expression pattern B_2 _in response to heroin (CHX blocked 106% of activation, 6% of activation below basal level, *P *< 0.001) and ethanol (CHX blocked 104% of activation, *P *< 0.001) (Figure 4d, e).

Microarray results indicated the inhibitory effects of corticotropin-releasing factor (CRF) receptor 1 (CRFR1) and GR antagonists (antalarmin and RU486, respectively, blocked 65% of ethanol activation, *P *< 0.05) on gene expression activation of the B_3 _subcluster (Figure 4f, g). RU486 also altered the expression of several B_1 _genes, for example, *Sgk1 *and *Plekhf1 *(Figure 4g). Therefore, the influence of GR receptor blockage on ethanol-induced expression of B_1_genes could not be correctly evaluated.

### Correlation with behavioral drug effects

To link the gene expression patterns with drug-related phenotypes, we analyzed the correlations between the transcriptional and behavioral drug effects in mice. Mutual interactions between the brain gene expression and behavioral profiles are complex and multidimensional. Therefore, it is difficult to define them using analyses performed with only the few available data points. However, even speculative results obtained from this analysis create the unique possibility of assigning different transcriptional alterations induced by various drugs to drug-related phenotypes. We observed a positive correlation of r = 0.62 (Pearson's method, *P *< 0.001) between the level of drug-induced locomotor activation and the degree of transcriptional response of gene expression pattern A. Additionally, we found a significant correlation between the acute induction of B_1 _genes and the rewarding effect of the drug (r = 0.7, Pearson's method, *P *< 0.05; Figure 1c, d; Additional file 9).

### Evaluation of two drug-regulated genes at the mRNA and protein levels

We selected two genes from expression pattern B for further evaluation. The first gene, S*gk1*, encodes the SGK protein (serum-and glucocorticoid-inducible kinase) and exhibited the B_1 _pattern. The second gene, *Tsc22d3*, encodes the GILZ protein (glucocorticoid-induced leucine-zipper protein) and exhibited the B_2 _pattern. We inspected alterations in mRNA abundance in the striatum during the acquisition and expression of the morphine-induced CPP. Both genes were induced 4 h after each of three subsequent sessions of morphine-induced (20 mg/kg i.p.) conditioning (between 1.5-fold and 3-fold over the control group). However, transcription of *Sgk1 *and *Tsc22d3 *was not activated during the behavioral expression of morphine-induced CPP (Figure 3b).

**Figure 5:**
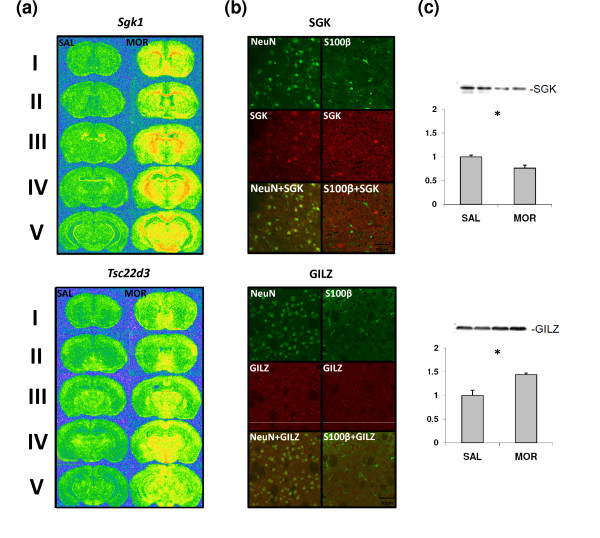
**Brain and cellular distribution of two selected drug-regulated genes**. **(a) **False-colored micrographs representing the relative level of the indicated mRNA 4 h after saline (SAL) or 20 mg/kg morphine (MOR) treatment revealed by *in situ *hybridization. Five coronal sections of mouse brain are presented, containing: (I) dorsal striatum and nucleus accumbens, (II) mid striatum, (III and IV) dorsal hippocampus and (V) ventral hippocampus/mesencephalon. **(b) **Confocal fluorescence micrographs showing coronal sections of striatum after immunohistochemical staining against SGK (*Sgk1*, red in the upper panel), GILZ (*Tsc22d3*, red in the lower panel), NeuN (neuronal marker, green, left) and S100B (glial marker, green, right). Scale bar: 50 μm. **(c) **Immunoblot of striatal lysates from mice 4 h after injection with morphine (MOR, 20 mg/kg i.p.) or saline (SAL) with antibodies against SGK and GILZ. The level of significance was measured using a two-tailed *t*-test: *P < 0.05. Error bars indicate standard error.

Furthermore, *in situ *hybridization was used to analyze the brain distribution of drug-induced changes in *Sgk1 *and *Tsc22d3 *expression. Both genes showed widespread induction throughout the brain, including the striatum. These results are in agreement with the microarray and qPCR data and confirm the strong striatal activation of both genes 4 h after morphine injection (20 mg/kg i.p.). More specifically, activation of *Tsc22d3 *in the striatum was limited to the medio-ventral region (nucleus accumbens), while *Sgk1 *was induced ubiquitously in the whole striatum (Figure 5a).

Western blotting was used to determine whether the changes in gene expression were translated into alterations in protein levels. The morphine-induced increase in *Sgk1 *abundance was associated with a significant decrease in the level of the protein (0.75-fold). Therefore, *Sgk1 *expression changes might be a compensatory effect to the loss of the protein. Up-regulation of *Tsc22d3 *was associated with an increase in the corresponding protein level (approximately 1.5-fold; Figure 5c). Double-immunofluorescence labeling with neuronal (NeuN) and astroglial (S100B) markers was used to identify cells that expressed SGK (*Sgk1*) and GILZ (*Tsc22d3*) proteins. In the mouse striatum, both genes appeared to be expressed mainly in neurons (Figure 5b).

### Drug-responsive genes are involved in the formation of dendritic spines

**Figure 6:**
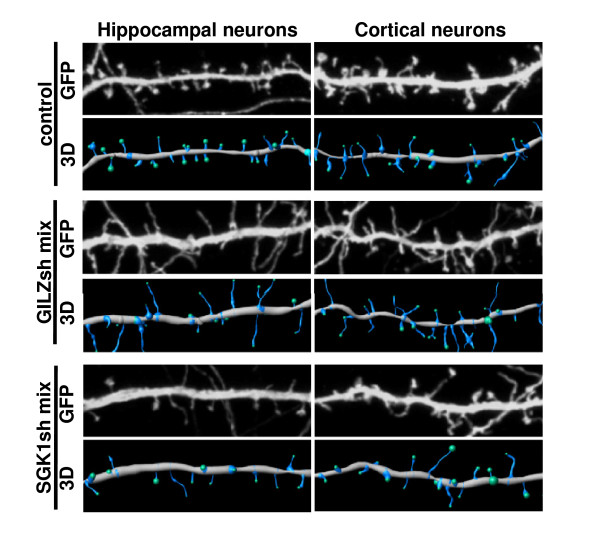
**The effects of *Tsc22d3 *and *Sgk1 *knockdown on dendritic spine morphology in cultured primary neurons**. Representative micrographs and three-dimensional Imaris reconstructions of dendritic segments of hippocampal and cortical neurons are presented. The neurons were transfected with pSUPER (control) or GILZsh mix or SGK1sh mix in pSUPER on day *in vitro *14 for 3 days. GFP was used to highlight transfected cell morphology.

In order to evaluate the roles of GILZ and SGK1 in neuronal plasticity, we knocked these proteins down in cultured primary neurons and analyzed the morphology of dendritic spines. To knock down our genes of interest, we first designed three and four short hairpin RNAs (shRNAs) against the *Tsc22d3 *and *Sgk1 *sequences, respectively, and cloned them into the pSUPER vector. This approach permits reliable and medium-term gene knockdown in neurons [55,56]. Next, we transfected hippocampal and cortical neuronal cultures for 3 days with mixes of shRNAs targeting each of the genes. The neurons were grown for 14 days before transfection because, at this stage, neuronal development is already completed and morphological changes can be attributed to spine plasticity. Co-transfection of green fluorescent protein (GFP) was used to identify and visualize the morphology of transfected cells. We used shRNA mixes to decrease the potential off-target effects of single hairpins and increase the probability of successful knockdown. As shown in Figure 6, cells transfected with control vector (pSUPER) displayed characteristics of mature neurons with a mushroom-type spine morphology. Transfection with the GILZ shRNA (GILZsh) mix, however, caused pronounced changes in spine morphology. Rather than mushroom-shaped spines, GILZsh-transfected neurons had thin, long, filopodia-like protrusions. On the other hand, transfection with the SGK shRNA (SGK1sh) mix did not cause pronounced changes in protrusion shape but resulted in a decrease in protrusion density compared to control neurons. Therefore, knockdown of GILZ or SGK1 in mature neurons resulted in changes in dendritic spine shape or density, respectively.

## Discussion

In this study, we aimed to define the sequence of molecular changes in the striatum in response to various drugs of abuse. We estimated that the number of genes induced by administration of the drugs of abuse was limited to approximately 100. Other studies reveal various numbers of genes with altered expression by drugs of abuse [26,41,44,46]. However, their estimations are based on arbitrary significance or fold-change thresholds, whereas our prediction was based on the number of true positives through a wide range of false discovery rates. The transcriptome changes produced by drugs of abuse were in contrast to alterations related to the diurnal cycle. While drugs of abuse produced few robust changes, the diurnal cycle alters the levels of several thousand transcripts (Additional file 1).

We found that almost all identified genes were regulated in concert with other genes in the form of two drug-responsive transcriptional modules. No other transcriptional modules were identified, even at lower significance thresholds. Therefore, we can assume that this study depicted all of the main patterns of induction in the mouse striatum after administration of drugs of abuse in rewarding doses. However, a recent study showed that after administration of higher, neurotoxic doses of methamphetamine, distinct gene expression patterns appear [40].

The first identified pattern (pattern A) consisted of IEGs, which are well described and commonly used as markers of neuronal activation [57-59]. Particular genes from this cluster were previously identified in the response to drugs of abuse, including *Fos*, *Fosb *and *Egr1 *[60,61]. Using bioinformatic analyses, we were able to postulate a role of the CREB and SRF transcription factors as main regulators of IEGs, which has been suggested previously [62,63]. Previous studies indicate that the cocaine-induced activity of CREB and SRF in the striatum is dependent on the D1R-downstream MEK/ERK signaling pathway [50,64]. Using pharmacological intervention, we established the role of D1R and the MEK/ERK signaling pathway for the whole group of IEGs. However, the role of a group of simultaneously expressed IEGs in neurons is not known. Some genes from pattern A (*Npas4, Homer1a *and *Arc*) may be involved in protecting against neuronal overexcitability [65,66]. *Npas4 *regulates inhibitory synapse development in an activity-dependent manner and diminishes the excitatory synaptic input neurons receive [67]. *Homer1a *appears to participate in the attenuation of the gradual inhibition of glutamate receptor-dependent calcium mobilization, as well as in mitogen-activated protein kinase activation [68]. Finally, increased expression of *Arc *may play a role in reducing AMPA receptor-mediated synaptic transmission [69,70]. We clearly demonstrated a lack of an effect of ethanol on the induction of IEGs in the striatum. It is worth mentioning that the induction of *Fos *(a member of pattern A) by cocaine is inhibited by ethanol [71-73]. We demonstrated that this lack of induction by ethanol was true for all IEGs. Therefore, the role of striatal IEGs in the development of ethanol addiction is rendered questionable.

The second identified pattern (pattern B) is relatively unknown. To our knowledge, this is the first comprehensive report describing the time- and drug-dependent induction of this gene expression pattern, although some of these genes have been previously reported by us and others [26,45,46]. This pattern consists of three subsequent sub-clusters (B_1_, B_2 _and B_3_). The examined drugs of abuse, with diverse pharmacological actions and behavioral effects, were all able to induce gene transcription of the relatively early pattern B_1_. Expression pattern B_1 _appeared to depend on several regulatory proteins, for example, transcription factors of the FOX family. The data also imply that patterns B_2 _and B_3 _appear to be regulated by steroid hormones that respond to morphine, heroin and ethanol. This is in agreement with activation of the hypothalamic-pituitary-adrenal axis (HPA) after administration of opioids and ethanol [74,75]. Our pharmacological dissection of drug-regulated gene expression profiles showed the inhibitory effects of CRFR_1 _and GR antagonists on ethanol-activated gene expression of the B_2 _and B_3 _subclusters. Therefore, the present results suggested that ethanol- and/or opioid-induced activation of these genes depends on HPA and the release of steroid hormones from the adrenal gland. This CRF- and GR-dependent signaling system is emerging as a key element of the neuroadaptive changes that are induced by drugs of abuse [76-78]. The identification of novel drug-responsive genes downstream of CRF/GR may help uncover the molecular mechanisms linking stress and addiction [79,80]. Pattern B genes modulate various aspects of cell functioning: hexose transport (*Slc2a1*), lipid metabolism (*Angptl4, Pparg*), regulation of sodium channels and the actin cytoskeleton (*Sgk1*) and regulation of the cell cycle (*Cdkn1a*), to mention just a few [81-84]. Our results clearly revealed qualitative and quantitative differences between the transcriptional networks affected by drugs of abuse. Therefore, it appears that various molecular mechanisms induced by drugs may lead to common addictive behaviors.

Previous studies show that cocaine administration leads to the release of glucocorticoids [85]. However, in our experiments, cocaine did not induce glucocorticoid-responsive genes (patterns B_2 _and B_3_). Expression of these genes was also affected relatively weakly by methamphetamine, compared to the much stronger effects of opioids and ethanol. Notably, both psychostimulants were the only drugs in our study that produced a prominent induction of IEGs (pattern A) within the first hour after drug injection. The pattern A genes included components of the transcription factor AP-1 (*Fos*, *Fosl2*, *Fosb*). Since AP-1 is a potent repressor of GR activity [86], it seems that GR-mediated effects of HPA activation on gene transcription in the striatum after psychostimulant administration may have been inhibited by previous induction of the AP-1 complex proteins due to D1R activation. Moreover, the identified clusters A and B contained their self-repressors. The IEG group contained ICER, an isoform of *Crem*, which acts as a powerful repressor of cyclic AMP-induced transcription [87]. Pattern B, which was partially controlled by glucocorticoids, includes *Fkbp5*, a chaperone that inhibits GR translocation into the nucleus [88].

HPA activation is one of the most recognized attributes of stress. Moreover, HPA activation is also a common effect of various drugs of abuse [74,75,85,89]. Chronic exposure to stress is associated with increased vulnerability to addiction [90,91]. The enhancing effects of stress on drug self-administration have also been documented in animal models [92]. Moreover, Mantsch *et al*. [93] show that corticosterone itself produces almost the same effects on drug taking as stress. Therefore, it is possible that corticosterone released after drug administration enhances the rewarding properties of the subsequent drug doses in the same way stress does. The mechanism of corticosterone contribution to addiction vulnerability is not well understood. Steroid-mediated enhancement of mesocorticolimbic dopamine neuron activity has been suggested to play a role [92].

A correlation analysis between the induction of gene clusters and the behavioral effects of particular drugs revealed that the induction of both B_1 _and B_2 _gene patterns may be associated with the rewarding effects of drugs. The present results suggested that two genes, representatives of patterns B_1 _(*Sgk1*) and B_2 _(*Tsc22d3*), might be associated with neuroplastic changes after administration of drugs of abuse. Some other authors have attempted to identify the role of *Sgk1 *in the central nervous system [94-96]. We demonstrated that knockdown of *Sgk1 *expression in neurons caused lower protrusion density and altered formation of dendritic spines [97]. GILZ (*Tsc22d3*) has already been considered in the context of its neuronal function [42,45,80]. However, we are the first to show that knockdown of *Tsc22d3 *provoked changes in spine morphology. Rather than mushroom-shaped spines, GILZsh-transfected neurons had thin, long, filopodia-like protrusions. These cellular changes may reflect an altered potential for neuronal plasticity and could be involved in the positive effect of corticosterone on vulnerability to addiction.

## Conclusions

We have identified two gene expression patterns that were induced in the striatum by various drugs of abuse and demonstrate that these patterns are the only ones induced by rewarding doses of these drugs. The gene patterns were not equally induced by the various drug classes. Therefore, clear differences between various drugs of abuse exist. We then identified upstream factors that control the discovered patterns. One of the patterns is at least partially controlled by HPA activation. We proposed a molecular mechanism that is involved in the HPA-activated enhancement of drugs' rewarding properties. Finally, we selected two genes and confirmed their influence on neuronal plasticity. In conclusion, this study provides valuable comparisons of the actions of various drugs of abuse on the striatal transcriptome and identifies potential target genes responsible for drug-induced neuroplasticity.

## Materials and methods

### Animals

Adult male (8 to 10 weeks old) C57BL/6J inbred mice were housed 6 to 10 per cage, under a 12-h dark/light cycle, with free access to food and water. Animals weighing 20 to 30 g were used throughout the experiments. The animal protocols used in the study were approved by the local Bioethics Commission at the Institute of Pharmacology, Polish Academy of Sciences (Krakow, Poland).

### Drug treatment

Mice were sacrificed by decapitation 1, 2, 4 or 8 h after a single morphine (20 mg/kg), heroin (10 mg/kg), ethanol (2 g/kg), nicotine (1 mg/kg), methamphetamine (2 mg/kg) or cocaine (25 mg/kg) i.p. injection, with respective saline and naïve control groups. The inhibitors and antagonists used in the secondary microarray experiment were i.p. injected 30 minutes before any of the drugs of abuse and were dissolved in an appropriate vehicle: 1 mg/kg SCH23390 (Biotrend, Koln, Germany) in saline; 30 mg/kg SL327 (Biotrend) in dimethyl sulfoxide (DMSO; Sigma-Aldrich, Steinheim, Germany); 40 mg/kg RU486 (Biotrend) in 3% Tween 20 (Sigma-Aldrich); 75 mg/kg cycloheximide (Biotrend) in saline; and 20 mg/kg antalarmin (Sigma-Aldrich) in 10% Cremophor EL (Sigma-Aldrich). Trichostatin (Sigma-Aldrich; 1 mg/kg in DMSO) injections were given 2 h before the cocaine. The doses of inhibitors/antagonists were based on the literature, paying particular attention to their ability to block drug-induced behavior.

### Behavioral testing

CPP tests were performed using an unbiased procedure in a three-arm apparatus. The experiment consisted of the following phases separated by 24 h: pre-conditioning test (day 0), conditioning with a drug dose as explained above (days 1, 3, 5), conditioning with saline (days 2, 4, 6) and post-conditioning test (day 7). For ethanol and nicotine, the procedure was prolonged to five sessions of conditioning. An independent group of animals was tested for drug-induced motor activation. Locomotor activity was measured in an activity cage in 15-minute intervals for 4 h following acute drug treatment.

### Tissue collection and RNA preparation

Samples containing the rostral part of the caudate putamen and the nucleus accumbens (referred to hereafter as the striatum) were collected. Tissue samples were placed in RNAlater reagent (Qiagen Inc., Valencia, CA, USA) and preserved at -70°C. Samples were thawed at room temperature and homogenized in 1 ml Trizol reagent (Invitrogen, Carlsbad, CA, USA). RNA was isolated following the manufacturer's protocol and further purified using the RNeasy Mini Kit (Qiagen Inc.). The total RNA concentration was measured using a NanoDrop ND-1000 Spectrometer (NanoDrop Technologies Inc., Montchanin, DE, USA). RNA quality was determined by chip-based capillary electrophoresis using an RNA 6000 Nano LabChip Kit and Agilent Bioanalyzer 2100 (Agilent, Palo Alto, CA, USA), according to the manufacturer's instructions. RNA from two mice was pooled to create a sample for each microarray.

### Gene expression profiling

A starting amount of 200 ng high-quality total RNA (equally pooled from two animals) was used to generate cDNA and cRNA with the Illumina TotalPrep RNA Amplification Kit (Illumina Inc., San Diego, CA, USA). The procedure consisted of reverse transcription with an oligo(dT) primer bearing a T7 promoter using Array-Script. The obtained cDNA became a template for *in vitro *transcription with T7 RNA polymerase and biotin UTP, which generated multiple copies of biotinylated cRNA. The purity and concentration of the cRNA were checked using an ND-1000 Spectrometer. Quality cRNA was then hybridized with Illumina's direct hybridization array kit (Illumina). Each cRNA sample (1.5 μg) was hybridized overnight to the MouseWG-6 BeadChip arrays (Illumina) in a multiple-step procedure according to the manufacturer's instructions; the chips were washed, dried and scanned on the BeadArray Reader (Illumina). Raw microarray data were generated using BeadStudio v3.0 (Illumina). Three biological replicates of the microarrays were prepared per experimental group. A total of 108 Illumina MouseWG-6 v1.1 and 84 Illumina MouseWG-6 v2 microarrays (with probes for approximately 48,000 transcripts) were used in the two experiments. To rule out the effects of injection and fluctuations related to circadian rhythms, we compared the drug effects to saline-treated and naïve animals. The microarray experimental design involved pooling two animals per array and combining three independent arrays per group. To provide an appropriate balance in the whole dataset, groups were equally divided between the array hybridization batches.

### Microarray data analysis

Microarray quality control was performed using BeadArray R package v1.10.0. The following parameters were checked on all 192 arrays: number of outliers, number of beads and percent of detected probes. After background subtraction, the data were normalized using quantile normalization and then log2-transformed. The obtained signal was taken as the measure of mRNA abundance derived from the level of gene expression. The results were standardized to reduce the effect of hybridization batches using z-score transformation. Statistical analysis of the results was performed using two-way ANOVA (for the factors drug and time) followed by Bonferroni correction for multiple testing. Alternatively, the FDR (percent FDR) was estimated using the Benjamini and Hochberg method [39]. To obtain drug-versus-saline comparisons, two-way ANOVA was followed by Tukey's post-hoc test. All statistical analyses were performed in R software version 2.8.1 [98]. Gene cross-annotation between the two versions of each microarray was performed automatically based on probe sequence, transcript ID and gene identifier, with some manual corrections.

### Cluster analysis

Hierarchical clustering was performed using the measure of Euclidian distance and average distance linkage methods. The cluster separation was performed according to an arbitrary threshold (h = 13). Several alternative clustering strategies produced similar hierarchical relationships, as shown in Figure 2a. Cluster visualization was performed using dChip software [99].

### Functional annotation, GO enrichment, cell type enrichment and literature mining

The functional annotation analysis tool DAVID 2008 was used to identify over-represented ontologic groups among the gene expression patterns and to group genes into functional classifications [100]. The list of transcripts represented on the Illumina Mouse WG-6 v1.1 microarray was used as a background list. Over-represented GO terms were defined as having at least three transcripts and *P *< 0.05 under Fisher's exact test. For cell-type enrichment of mRNA, a recently published brain transcriptome database was used [101]. The database of 1267 gene lists was used for the literature enrichment analysis. This included gene lists manually extracted from the published data, as well as a collection of gene sets from the MSigDB database [29]. The statistical significance analysis of transcript enrichment was performed using the ORA algorithm in ErmineJ software [102]. Annotation handling was based on Mouse Gene Symbol IDs (MGI), and all other annotation formats were translated using BioMart [103]. Input data, annotations and the obtained results are included in Additional files 2, 7 and 8.

### Identification of transcription factor binding sites enriched in co-regulated transcripts

The identification of over-represented TFBSs was performed using the cREMaG database [104] with default parameters. Briefly, a 70% conservation threshold and a maximum number of 50 conserved TFBSs in non-coding regions between 5,000 bp upstream and 1,000 bp downstream of the transcriptional start site were used. Functional promoter sequences were identified by alignments between 5' upstream sequences of mouse and human orthologous genes. The identification of TFBSs was performed using the Perl TFBS module and matrices from the JASPAR database [105]. MGI Gene Symbol lists were submitted, and default parameters were used.

### Validation of microarray data by qPCR

We performed qPCR measurements for a set of genes representative of the identified gene clusters. Reverse transcription was performed with Omniscript Reverse Transcriptase enzyme (Qiagen) at 37°C for 60 minutes. The reaction was carried out in the presence of the RNase inhibitor rRNAsin (Promega, Madison, WI, USA), and an oligo(dT16) primer (Qiagen) was used to selectively amplify mRNA. qPCR reactions were performed using Assay-On-Demand TaqMan probes (Additional file 3) according to the manufacturer's protocol (Applied Biosystems, Foster City, CA, USA) and were run on an iCycler (Bio-Rad, Foster City, CA, USA). For each reaction, approximately 50 ng of cDNA synthesized from a total RNA template (isolated from an individual animal) was used (n = 4 to 10). To minimize the contribution of contaminating genomic DNA, primers were designed to span exon junctions. Additionally, control reactions without reverse transcription enzyme for each assay were performed. The amplification efficiency for each assay was determined by running a standard dilution curve. The expression of the *Hprt1 *(hypoxanthine guanine phosphoribosyl transferase 1) transcript, which had a stable mRNA level, was quantified to control for variations in cDNA levels. The cycle threshold values were calculated automatically by iCycler IQ 3.0a software with default parameters. The abundance of RNA was calculated as 2^-(threshold cycle)^.

### Measurement of the effects of pharmacological dissection

Further microarray experiments were performed to analyze the effects of selected pharmacological tools on drug-induced gene expression changes. Mean fold changes of drug-induced transcriptional activation for each gene expression pattern with and without the administration of a particular inhibitor or antagonist were compared. The influence was measured as a percentage of inhibition of the drug-induced transcriptional response, with 0% representing no effect and 100% representing complete inhibition. The statistical significance of influence was measured as a comparison of the mean fold change between the drug plus inhibitor/antagonist and saline plus vehicle versus drug plus vehicle and saline plus vehicle groups. All necessary controls, including different vehicles and time points, were included.

### Association of gene expression patterns with phenotype

The correlation between the effects of the drugs and behavioral effects in animals was measured using Pearson's method. The mean expression change of each gene was summarized for all time points together for correlation with CPP and for single time points for correlation with locomotor activation.

### Western blotting

Protein was extracted from the samples using RIPA buffer. The protein concentration of each sample was determined using the BCA Protein Assay Kit (Sigma-Aldrich). Aliquots of crude extracts (containing 5 to 20 μg of protein) were then subjected to electrophoresis on a 12% SDS-polyacrylamide gel, and proteins were electroblotted onto microporous polyvinylidene difluoride (PVDF) membranes (Roche, Germany). The membranes were blocked for 1 h, washed and incubated overnight with primary antibodies at 4°C. After washing, immunocomplexes were detected using a Chemiluminescence Western Blotting Kit (Mouse/Rabbit, Roche), visualized with a Fujifilm LAS-1000 fluoroimager system and quantified using Image Gauge software (Fujifilm, Tokyo, Japan). For immunoblotting, a rabbit polyclonal antibody raised against: a synthesized non-phosphopeptide derived from human SGK1 around the phosphorylation site of serine 78 (P-P-S^P^-P-S; Abcam, Cambridge, MA, USA); or a synthetic peptide conjugated to keyhole limpet hemocyanin derived from residue 100 to the carboxyl terminus of Mouse GilZ/TilZ (Abcam) was used. To control for transfer quality, each PVDF blot was stained with Ponceau S.

### *In situ *hybridization

The frozen brains were cut into 12-μm-thick coronal sections on a cryostat microtome CM 3050S (Leica Microsystems, Germany), and the sections were thaw-mounted on gelatin-chrome alum-coated slides and processed for *in situ *hybridization. The hybridization procedure was performed as previously described [106]. Briefly, the sections were fixed with 4% paraformaldehyde, washed in PBS and acetylated by incubation with 0.25% acetic anhydrite (in 0.1 M triethanolamine and 0.9% sodium chloride). The sections were then dehydrated using increasing concentrations of ethanol (70 to 100%), treated with chloroform for 5 minutes and rehydrated with decreasing concentrations of ethanol. The sections were hybridized for 15 h at 37°C with oligonucleotide probes complementary to nucleotides 493-536 of the mouse *Tsc22d3 *cDNA (5'-CAGTTGCTCGGGGCTTGCCAGCGTCTTCAGGAGGGTGTTCTCGC-3'; NM 010286.3) and nucleotides 1682-1725 of the mouse *Sgk1 *cDNA (5'-TTGATCACAGCTCAGACAGACTGCGGGGATTCCTCTTAGACCTG-3'; NM 011361.1). The probes were labeled with ^35^S-dATP by the 3'-tailing reaction using terminal transferase (MBI Fermentas, Vilnius, Lithuania). After hybridization, the slices were washed three times for 20 minutes with 1×SSC/50% formamide at 40°C and twice for 50 minutes with 1×SSC at room temperature. Then, the slices were dried and exposed to phosphorimager plates (Fujifilm) for 5 days. The hybridization signal was digitized using a Fujifilm BAS-5000 phosphorimager and Image Reader software.

### Immunohistochemistry

The animals were deeply anesthetized (pentobarbital, 60 mg/kg i.p.) and perfused transcardially with saline followed by 4% paraformaldehyde in 0.1 M phosphate buffer, pH 7.4. Brains were removed, postfixed for 4 h, transferred to PBS and stored at 4°C. Free-floating sections were cut 40-μm thick using a Leica vibratome. For double-immunofluorescence labeling, sections were blocked for 1 h in 5% donkey serum, pH 7.4 (Vector Labs, Burlingame, CA, USA) and then incubated overnight at 4°C in a mixture of primary antibodies. Respective pairs of antibodies included rabbit polyclonal anti-GILZ (1:100; Abcam) or rabbit polyclonal anti-SGK1 (1:400; Abcam) with mouse monoclonal anti-s100-beta (1:1,500; Sigma-Aldrich) or mouse monoclonal anti-NeuN (1:250; Chemicon, Rosemont, IL, USA). After three washes in PBS, double immunofluorescence was revealed by incubating the sections for 2 h at room temperature in a mixture of secondary antibodies: Alexa Fluor 488-conjugated donkey anti-mouse IgG and Alexa Fluor 555-conjugated donkey anti-rabbit IgG (both at 1:750; Molecular Probes Inc., Eugene, OR, USA). The sections were washed three times with PBS, mounted on slides in Vectashield (Vector Labs) and coverslipped. The negative controls were prepared by omitting the primary antibody. The sections were examined using a 63× objective on a confocal microscope (DMRXA2 TCS SP2, Leica Microsystems). The background noise of each confocal image was reduced by averaging four scans per line and four frames per image. To visualize image details, plates were generated adjusting the contrast and brightness of digital images (ImageJ, NIMH).

### Primary neuron cultures and transfection

Primary hippocampal and cortical cultures were prepared from embryonic day 16 mouse brains, according to the Banker and Goslin procedure. Cells were plated on coverslips coated with poly-L-lysine (30 μg/ml; Sigma) and laminin (2 μg/ml; Roche) at a density of 500 (hippocampal) or 1,250 (cortical neurons) cells/mm^2^. Neuronal cultures were grown in Neurobasal medium (Invitrogen) supplemented with B27 (Invitrogen), 0.5 mM glutamine, 12.5 μM glutamate and penicillin/streptomycin mix (Sigma). On the 14th day *in vitro *(DIV), neurons were transfected with Lipofectamine 2000 (Invitrogen) for 3 days, as previously described [55]. Briefly, for cells growing in a single well of a 24-well dish, 0.9 μg of DNA was mixed with 1.67 μl of Lipofectamine 2000 in 100 μl of Neurobasal medium and incubated for 30 minutes. During the incubation time, fresh culture media were prepared, mixed half and half with old media and split into two equal aliquots. The first aliquot was added to the cells, and the second was saved during the transfection period. Next, complexes of DNA with Lipofectamine 2000 were added to the cells and incubated for 4 h at 37°C, 5% CO_2_. Finally, cells were washed twice with Neurobasal medium and incubated in the saved culture media. The pSUPER vector [107] and β-actin-GFP [56] mammalian expression plasmids have been described. GILZ shRNAs were designed against mouse GILZ (*Tsc22d3*) mRNA (EntrezGene ID: 14605) targeting nucleotides 383-401, 385-407 and 437-459, respectively. SGK1 shRNAs targeted nucleotides 265-283, 719-737, 975-993 and 1008-1026 of *Sgk1 *(EntrezGene ID: 20393), respectively. All shRNAs were cloned into pSUPER. In RNA inyerference experiments, the mix of shRNAs encoding plasmids and β-actin-GFP were cotransfected at a 3:1 ratio.

### Immunocytochemistry of *in vitro *cultured neurons

For immunofluorescent staining of transfected GFP, neurons were fixed with 4% paraformaldehyde containing 4% sucrose in PBS for 10 minutes at room temperature. After fixation, cells were washed three times with PBS for 5 minutes at room temperature and incubated with primary antibody in GDB buffer (0.2% gelatin, 0.8 M NaCl, 0.5% Triton X-100 and 30 mM phosphate buffer, pH 7.4) overnight at 4°C. Cells were then washed three times with PBS for 10 minutes at room temperature. Secondary antibodies were applied in GDB for 1 h at room temperature and washed out by three 10-minutes PBS washes. The secondary antibodies were rabbit anti-GFP (Medical and Biological Laboratories Co. Ltd., Nagoya, Japan) and anti-rabbit Alexa Fluor 488-conjugated secondary antibody (Invitrogen). Confocal images of cells were obtained with sequential acquisition settings at the maximal 1,024 × 1,024 pixel resolution of the Zeiss LSM 5 Exciter microscope (Carl Zeiss, Germany). Each image was a z-series of images, and each was averaged two times. The obtained stacks were directly analyzed using Imaris v6.3.1 (Bitplane AG, Zurich, Switzerland). The changes in spine length, diameter and volume were quantified based on the three-dimensional reconstructions computed by Imaris.

### Regulatory network modeling

**Figure 7:**
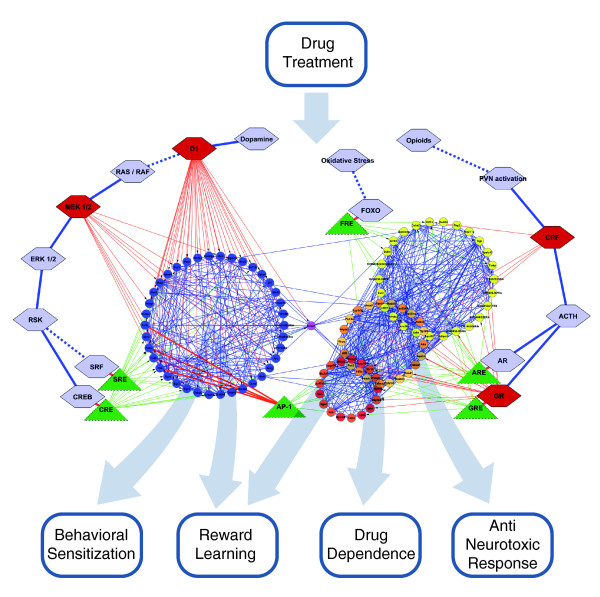
**A proposed scheme of the core regulatory network of drug-induced molecular mechanisms and gene expression alterations in the striatum**. Small nodes represent transcripts belonging to the identified gene expression patterns. The color of each node reflects its gene pattern membership: blue, A; yellow, B_1_; orange, B_2_; and red, B_3_. Thin blue edges between the nodes indicate a correlation between the expression profiles of two genes. Functional connections were implemented based on our results from literature mining, pharmacological experiments and *in silico *predictions of TFBSs. Large hexagonal nodes represent elements of drug-activated signaling pathways. Solid and dashed edges between the nodes indicate direct or indirect interactions, respectively, as suggested by the literature. A red node color and thin red edge indicate a pharmacologically verified connection (Figure 4). Green triangle nodes represent gene transcription regulatory elements. Thin green edges indicate positive detection of TFBSs in a promoter region of a particular gene. Transparent arrows suggest the influence of gene expression changes on addiction-related traits based on the correlations between the transcriptional and phenotypic response (Figure 1c, d; Additional file 9).

Putative drug-activated signaling pathways and interactions between the transcription factors and drug-responsive genes were modeled. Data were integrated based on our results from gene expression profiling, pharmacological experiments and *in silico *predictions of TFBSs. Only strong correlations (r > 0.6) between the expression profiles of two genes and pharmacologically verified connections (*P *< 0.1) were analyzed. The main construction of signaling pathways was based on the literature. To generate the molecular network shown in Figure 7, we used Cytoscape software.

### Accession codes

Microarray data were submitted to the NCBI Gene Expression Omnibus (GEO) under accession number [GEO:GSE15774].

## Abbreviations

bp: base pair; CHX: cycloheximide; CPP: conditioned place preference; CREB: cyclic AMP-response element binding protein; CRF: corticotrophin-releasing factor; CRFR1: corticotrophin-releasing factor receptor 1; D1R: dopamine receptor 1; DMSO: dimethyl sulfoxide; ERK1/2: extracellular signal-regulated kinase; FDR: false discovery rate; GFP: green fluorescent protein; GO: Gene Ontology; GR: glucocorticoid receptor; HPA: hypothalamic-pituitary-adrenal axis; IEG: immediate early gene; i.p.: intraperitoneally; PBS: phosphate-buffered saline; PVDF: polyvinylidene difluoride; qPCR: quantitative PCR; shRNA: short hairpin RNA; SRF: serum response factor; TFBS: transcription factor binding site.

## Authors' contributions

MP and MK carried out the microarray data analysis. MK and MP designed the study, interpreted the results and wrote the manuscript. MP performed functional annotation, literature mining and analysis of TFBSs. MK and EK carried out qPCR. WS was responsible for drug treatment and behavioral testing. WB and MS performed protein analyses. AG and BZ conducted *in situ *hybridization experiments. MS, IC, LS and JJ provided data from gene knockdown experiments. RP coordinated the study, carried out tissue preparation and reviewed the manuscript. All authors read and approved the final manuscript.

## Supplementary Material

Additional file 1 A figure presenting ANOVA results of gene expression profiling of drug effects in mouse striatum. The upper panel shows the relationship between the number of true positive results and the proportion of false positives for **(a) **time and **(b) **drug factors and **(c) **their interaction in ANOVA. The lower panel presents the relationship between the obtained *P*-values (y-axis) for both the factors and their interaction and the theoretically expected *P*-values (x-axis). Click here for file

Additional file 2 A table listing the results of two-way ANOVA (followed by correction for multiple comparisons or Tukey's post-hoc test). The lists include: those genes altered by drug treatment, by time and with interaction between the factors; those genes regulated by each particular drug (*P *< 0.05, versus saline); and the expression levels of genes from patterns A and B. Each of these is available as a separate spreadsheet. *P*-values obtained from two-way ANOVA were further corrected using Bonferroni or Benjamini and Hochberg (percent FDR) corrections. Click here for file

Additional file 3 A data file providing the results from the qPCR validation of the microarray data. Results for selected genes are presented as the mean (± standard error) compared with the saline control group (n = 3 to 10). A list of TaqMan assays used in the qPCR experiments with IDs and exon boundaries is included as a separate sheet. Click here for file

Additional file 4 A figure showing hierarchical clustering of drug-induced gene expression alterations in mouse striatum. Microarray results are shown as a heat map and include genes with a significance obtained from two-way analysis of variance of the drug factor at **(a) **5% and **(b) **29% of FDR. Colored rectangles represent the transcript abundance (Additional file 5) of the gene and are labeled on the right. The intensity of the color is proportional to the standardized values (between -2 and 2) from each microarray, as indicated on the bar below the heat map image. Click here for file

Additional file 5 A figure showing chromosome localizations of drug-responsive genes. Click here for file

Additional file 6 A figure showing comparison of drug-induced effects in mouse striatum. (**a-g**) Average activity of time-dependent, drug-induced gene expression patterns. The results are presented as mean changes in gene expression (measured using z-values, in the extended A, B_1_, B_2 _and B_3 _groups of genes). The values are relative to the level of transcript abundance in naïve animals (at each of the time points 1, 2, 4 and 8 h). The thickness of the line is proportional to the number of genes in each cluster. **(h,i) **Matrices of correlation between all compared drug-induced gene expression profiles. The results were obtained using **(h) **DNA microarrays and **(i) **qPCR. The qPCR analysis was used to validate microarray results (Additional file 3). Click here for file

Additional file 7 A table listing the complete results of the GO analysis presented in the manuscript. The analyses were performed on lists of genes that correspond to patterns A, B_1_, B_2 _and B_3_. The genes are listed in Additional file 2. Selected results are presented in Table 1. Click here for file

Additional file 8 A table listing the complete results of the literature mining presented in the manuscript. The analyses were performed on lists of genes that correspond to patterns A, B_1_, B_2 _and B_3_. The genes are listed in Additional file 2. Selected results are presented in Table 1. Click here for file

Additional file 9 A table providing the results of correlation analysis between the transcriptional response to drugs of abuse and behavioral traits related to drug abuse (see Materials and methods). Behavioral data and the matrix of correlations are available as separate sheets. Gene expression data from each pattern were normalized using z-score transformation and summarized as a function of time. Associations were computed using Pearson's correlation. Click here for file
