# Supplementary figures and images for: The dissection of transcriptional modules regulated by various drugs of abuse in the mouse striatum
Source: Genome Biol. 2010 May 4;11(5):R48. doi: 10.1186/gb-2010-11-5-r48 (PMC2898085; doi:10.1186/gb-2010-11-5-r48)

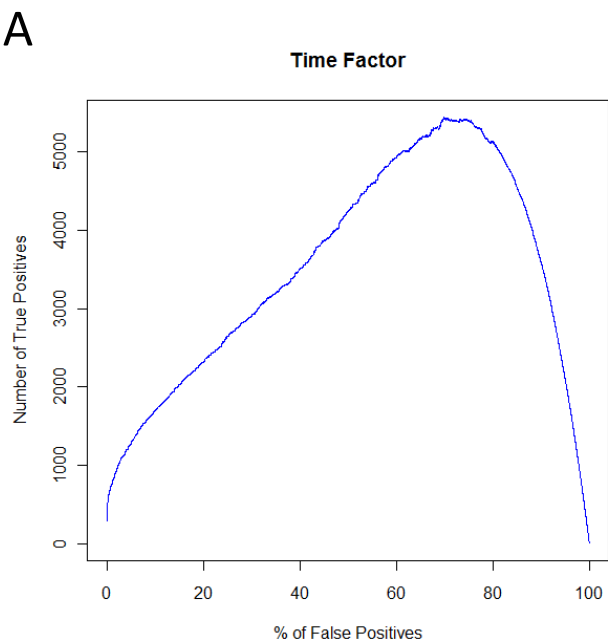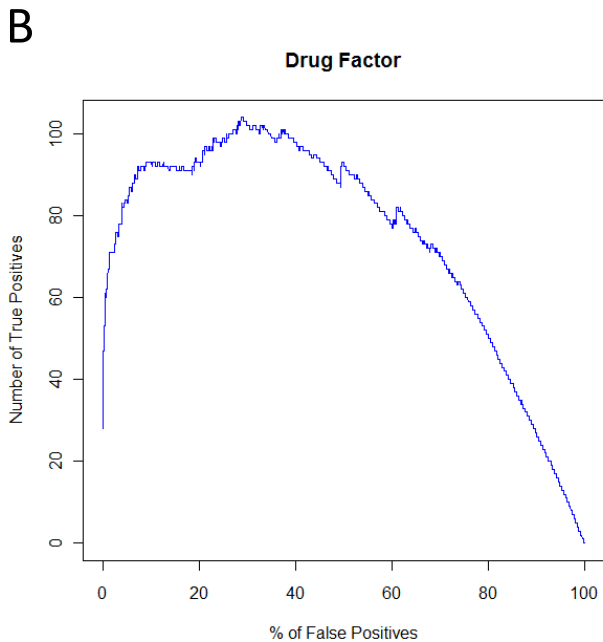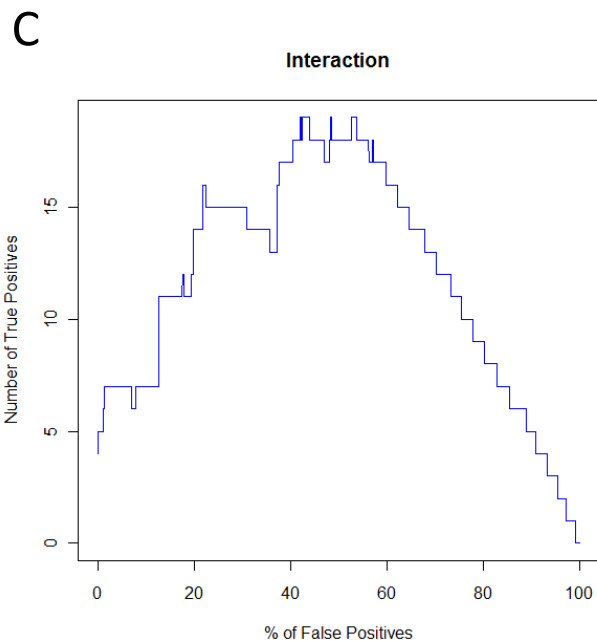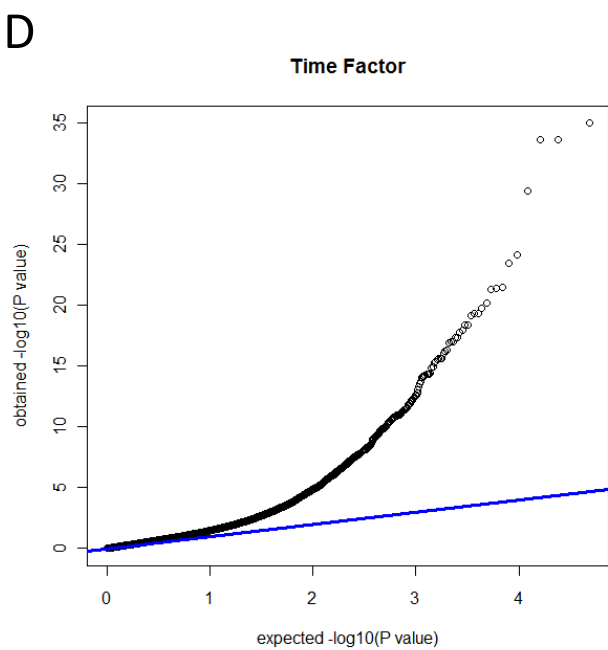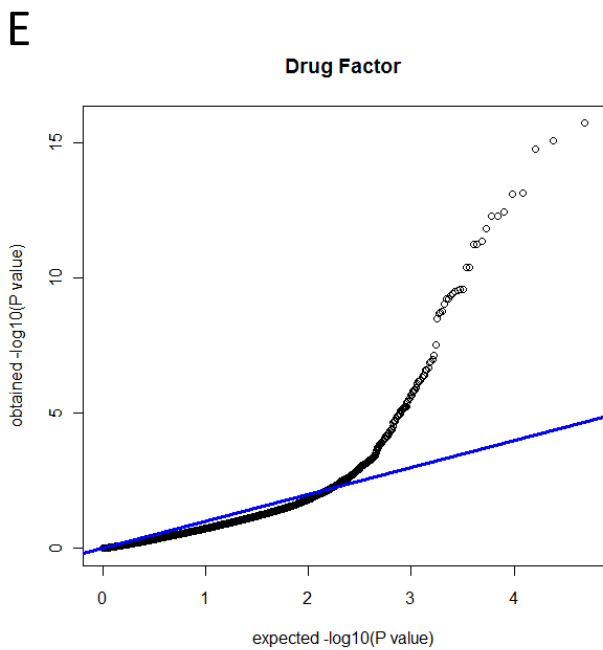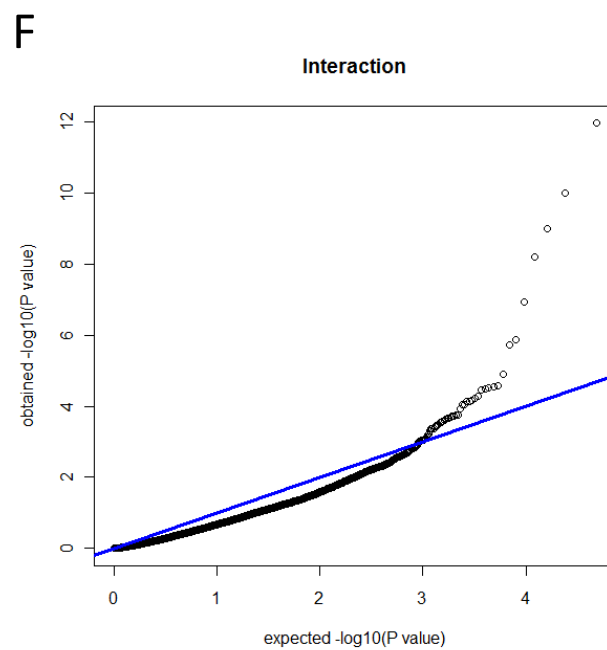

Supplement: Additional file 1 — A figure presenting ANOVA results of gene expression profiling of drug effects in mouse striatum. The upper panel shows the relationship between the number of true positive results and the proportion of false positives for (a) time and (b) drug factors and (c) their interaction in ANOVA. The lower panel presents the relationship between the obtained P-values (y-axis) for both the factors and their interaction and the theoretically expected P-values (x-axis). [file gb-2010-11-5-r48-S1.PDF]

A

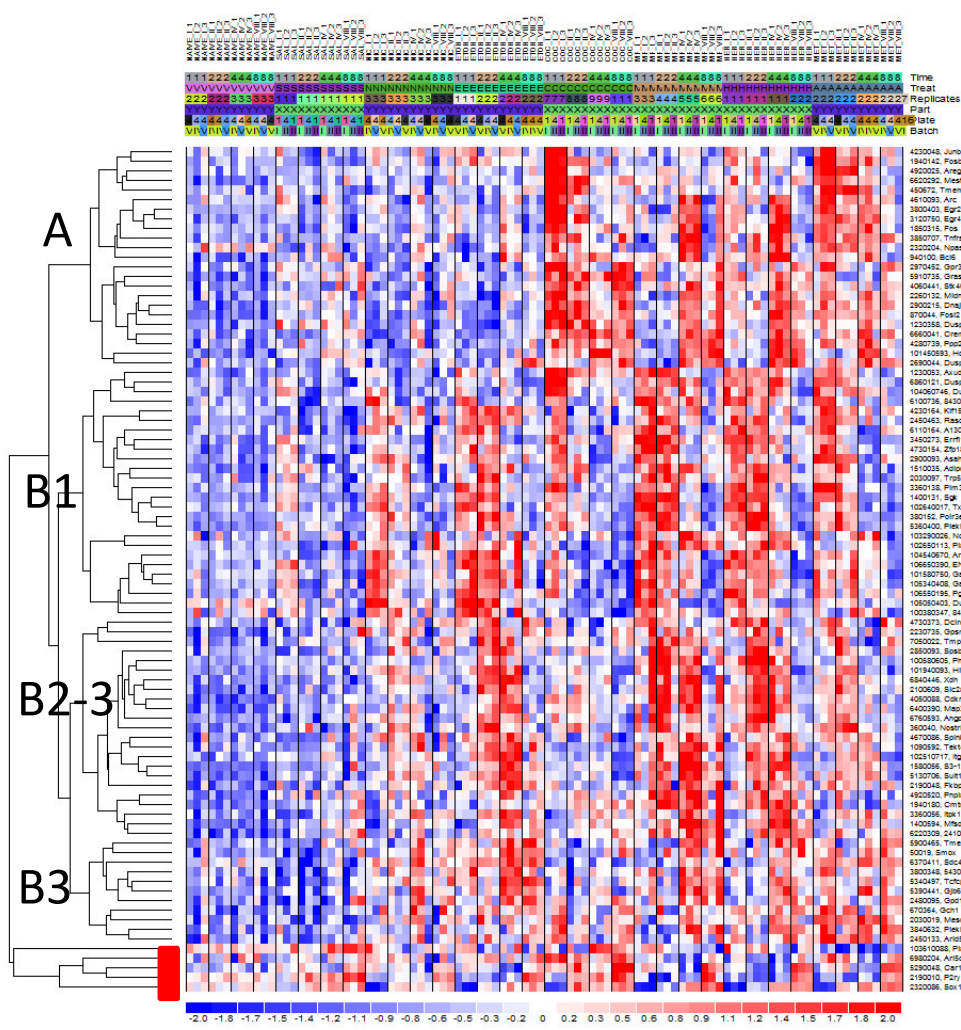

B

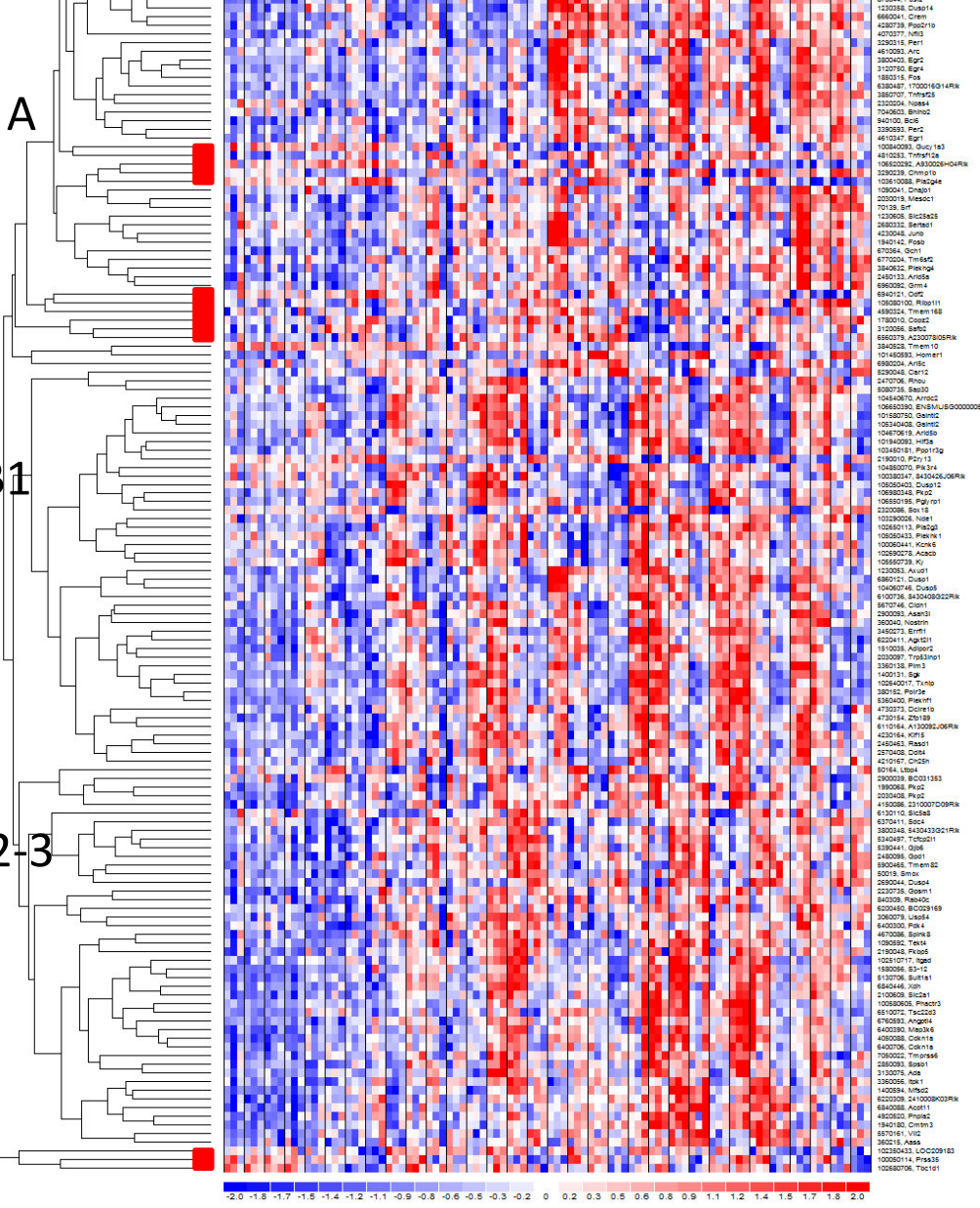

Supplement: Additional file 4 — A figure showing hierarchical clustering of drug-induced gene expression alterations in mouse striatum. Microarray results are shown as a heat map and include genes with a significance obtained from two-way analysis of variance of the drug factor at (a) 5% and (b) 29% of FDR. Colored rectangles represent the transcript abundance (Additional file 5) of the gene and are labeled on the right. The intensity of the color is proportional to the standardized values (between -2 and 2) from each microarray, as indicated on the bar below the heat map image. [file gb-2010-11-5-r48-S4.PDF]

# Mouse Genome

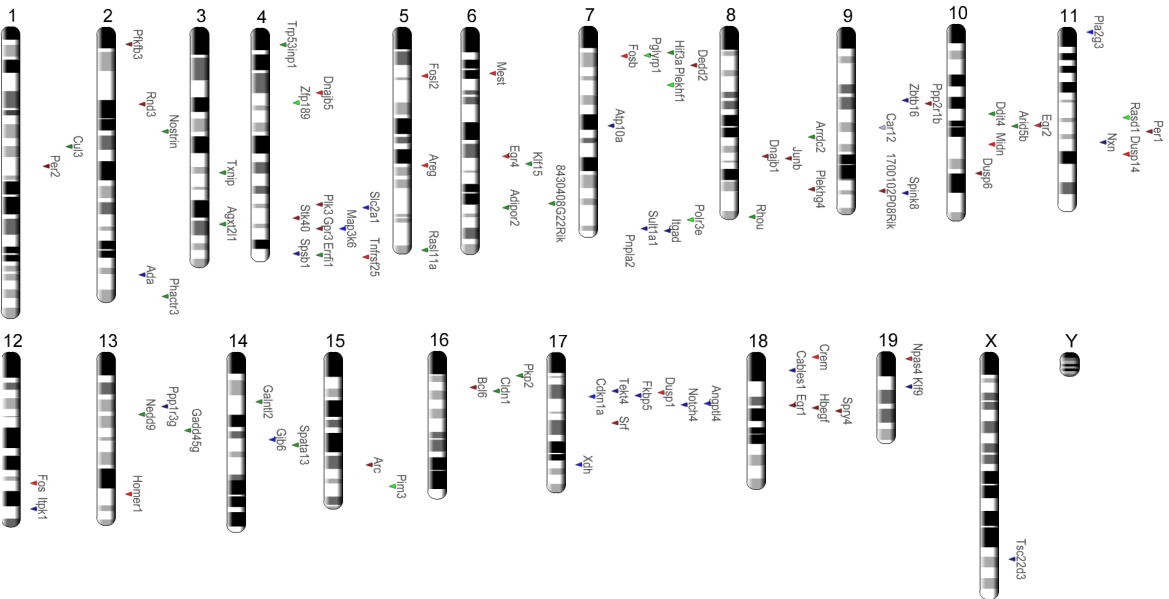

Supplement: Additional file 5 — A figure showing chromosome localizations of drug-responsive genes. [file gb-2010-11-5-r48-S5.PDF]

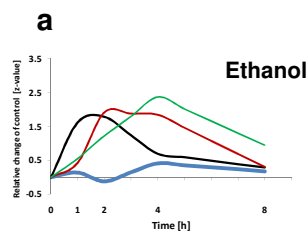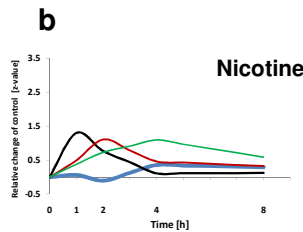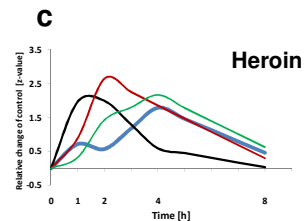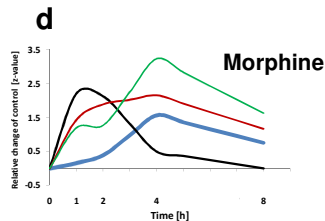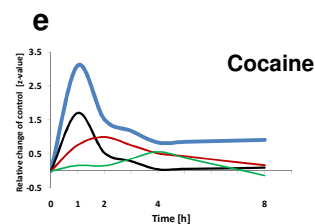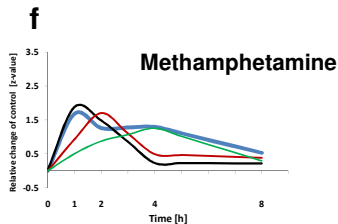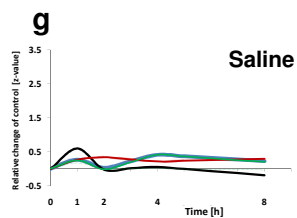

— pattern A  
— pattern B<sub>1</sub>  
— pattern B<sub>2</sub>  
— pattern B<sub>3</sub>

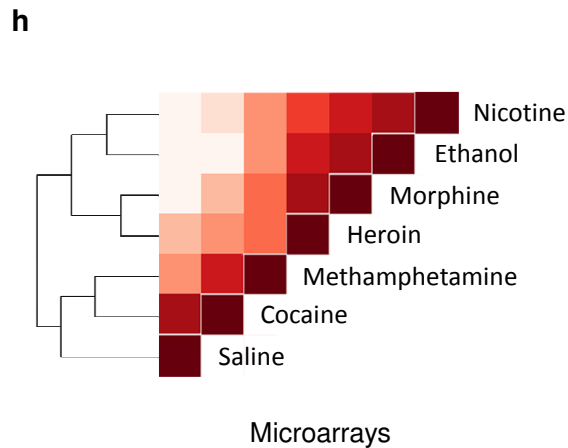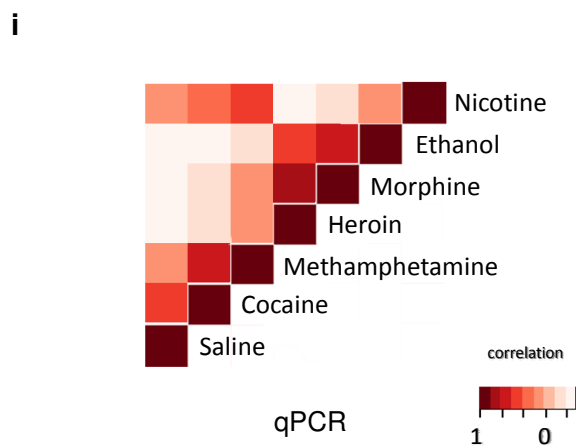

Supplement: Additional file 6 — A figure showing comparison of drug-induced effects in mouse striatum. (a-g) Average activity of time-dependent, drug-induced gene expression patterns. The results are presented as mean changes in gene expression (measured using z-values, in the extended A, B1, B2 and B3 groups of genes). The values are relative to the level of transcript abundance in naïve animals (at each of the time points 1, 2, 4 and 8 h). The thickness of the line is proportional to the number of genes in each cluster. (h,i) Matrices of correlation between all compared drug-induced gene expression profiles. The results were obtained using (h) DNA microarrays and (i) qPCR. The qPCR analysis was used to validate microarray results (Additional file 3). [file gb-2010-11-5-r48-S6.PDF]
